# Stream invertebrate communities of Mongolia: current structure and expected changes due to climate change

**DOI:** 10.1186/2046-9063-8-18

**Published:** 2012-08-24

**Authors:** Alain Maasri, Jon Gelhaus

**Affiliations:** 1 The Academy of Natural Sciences of Drexel University, 1900 Ben Franklin Parkway, Philadelphia, PA 19103-1195, USA

**Keywords:** Macroinvertebrates, Nestedness, Hydrological connectivity, Conservation, Central Asia, Aquatic insects

## Abstract

**Background:**

Mongolia’s riverine landscape is divided into three watersheds, differing in extent of permafrost, amount of precipitation and in hydrological connectivity between sub-drainages. In order to assess the vulnerability of macroinvertebrate communities to ongoing climate change, we consider the taxonomic and functional structures of stream communities in two major watersheds: The Central Asian Internal Watershed (CAIW) and the Arctic Ocean Watershed (AOW), together covering 86.1% of Mongolia’s surface area. We assess the consequences of the hydrological connectivity between sub-drainages on the nestedness and distinctness of the stream communities. And accordingly, we discuss the expected biotic changes to occur in each watershed as a consequence of climate change.

**Results:**

Gamma and beta diversities were higher in the CAIW than the AOW. High community nestedness was also found in the CAIW along with a higher heterogeneity of macroinvertebrate assemblage structure. Assemblages characteristic of cold headwater streams in the CAIW, were typical of the drainages of the Altai Mountain range. Macroinvertebrate guilds of the CAIW streams exhibited traits reflecting a high stability and low resilience capacity for eutrophication. In contrast, the community of the AOW had lower nestedness and a combination of traits reflecting higher stability and a better resilience capacity to disturbances.

**Conclusion:**

Higher distinctness of stream communities is due to lower connectivity between the drainages. This was the case of the stream macroinvertebrate communities of the two major Mongolian watersheds, where connectivity of streams between sub-drainages is an important element structuring their communities. Considering differences in the communities’ guild structure, hydrological connectivity and different magnitudes of upcoming impacts of climate change between the two watersheds, respective stream communities will be affected differently. The hitherto different communities will witness an increasing differentiation and divergent adaptations for the upcoming changes. Accordingly, in an increasing awareness to protect Mongolia’s nature, our results encourage adapting conservation planning and management strategies specifically by watershed.

## Background

Over the last two decades human-caused climate change has emerged as a leading threat to biodiversity conservation [1-3]. The projected consequences of climate change on the aquatic biota have been a concern of both conservation biologists [4] and ecologists [5] and often showing a rather grim future. Climate change scenarios forecast global increases of temperature, annual precipitation, and atmospheric moisture [6] along with the degradation and shrinkage of permafrost [7]. These modifications will have severe consequences on freshwater ecosystems [8] and are expected to increase threats on the conservation of freshwater biodiversity [9]. Climate change is expected to operate on two different spatial scales [10]. First, on a local scale, the modification of thermal and hydrological regimes may alter biotic interactions such as competitive behavior, population dynamics and energy fluxes. Second, on a regional scale, abiotic forcing may lead to changes in constraints on species distribution, leading to fragmented biotas and reshaped niches. Given the important role of niche conservation in climatic tolerances, species ranges are expected to shift [see, [11] to track climatic regimes to which they are adapted. Intolerant species or those with limited dispersal ability may be at risk of extinction. Given the rapid rate of climate change, evolutionary adaptation is less likely to occur, while the phenotype plasticity of species will play the key role in preserving populations.

To predict the response of communities to climate change, we need first to understand how communities are currently structured and regulated. The structure of a stream invertebrate community is the result of several multiscale filters representing both historical factors and ecological constraints. The historical factors are considered to operate on a larger geographical scale and over geological time periods; while the ecological constraints are considered to operate mainly on the habitat scale and on shorter time scales [12,13]. The dynamics of a stream invertebrate community is highly dependent on the structure and diversity of its guilds. Guild ecology examines communities by assigning each species to a functional guild (i.e., groups of species that have common biological traits). Therefore, plasticity of species phenotype and most probably intra-guild species diversity will have major influences on the regulation and conservation of a community as a functional unit. Guild ecology has considerably increased our understanding of community organization and functioning [12,14,15] and has been proven reliable to study climate change scenarios [12,16].

The impact of climate change on stream macroinvertebrate communities has been mainly studied in Europe [16] and North America [5]. No large-scale studies have been carried out in Central Asia, although global warming is severely affecting this region and particularly Mongolia (see below for details). In this paper, 1) we describe the taxonomic and functional structures of the stream macroinvertebrates communities of the two major watersheds of Mongolia (see below for details). These communities are to be used as a baseline to assess potential upcoming changes facing the Mongolian stream ecosystem. 2) Given the major differences in the hydrological connectivity of drainages between the studied watersheds: we predict lower community distinctness due to higher dispersal routes in the watershed having connected sub-drainages, versus more distinctness in the watershed having isolated sub-drainages. Hydrological connectivity is used here in an ecological sense to refer to water-mediated transfer of organisms [17] among the different sub-drainages of the watershed (see below for details). Accordingly, 3) we discuss the expected changes to occur, in the communities of each watershed and their resilience capacities [sensu, [18].

## Methods

### Study region

**Figure 1:**
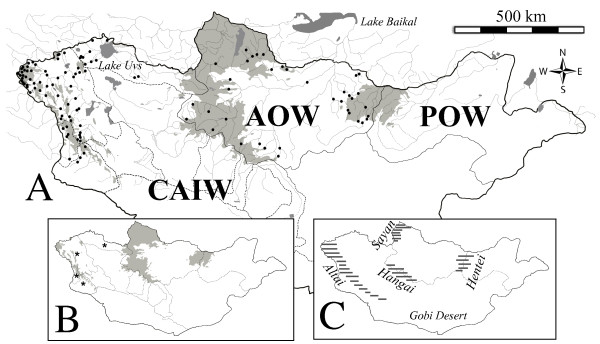
**Map showing the Mongolian watersheds (Figure 1A) with the different drainages (light gray lines) and standing water-bodies (dark gray areas). **The bold circles (Figure1**A**) are the locations of the sampled stream-sites included in this study. Figure 1**A** shows the three Mongolia watersheds separated by a bold lines (same bold lines are shown in Figure 1**C**), AOW is for the Arctic Ocean Watershed, CAIW is for the Central Asian Internal Watershed and POW for the Pacific Ocean Watershed. The dashed lines (Figure 1**A**, CAIW watershed) show the 11 sub-drainages of the CAIW, four of them sampled in this study (marked by asterisk in Figure 1**B**). The light gray shading (Figure 1**A**, 1**B**) shows the permafrost extent (above 50% permafrost content). Figure 1**C** shows the location of the major mountain ranges in Mongolia (dashed areas), and the location of the Gobi Desert.

Mongolia’s riverine landscape is structured in three watersheds (Figure 1), two of them are open watersheds and drain into the Arctic Ocean and the Pacific Ocean, respectively; while, the third is a closed watershed and drains into Central Asia. The studied region includes drainages of the Arctic Ocean Watershed (AOW) and the Central Asian Internal Watershed (CAIW) covering a surface area equivalent to 86.2% of Mongolia (equivalent to 1,438,267.7 km^2^). The AOW is bounded by Russia to the north, by the Hentei Mountain range to the east and by the Hangai Mountain range to the south and west. The CAIW is also bounded by Russia to the north, and is separated from China by the Gobi Desert to the south (Figure 1C).

The two studied watersheds are profoundly different:

1) The AOW covers only 20.6% of Mongolia’s surface area yet receives 51% of the annual precipitation of Mongolia while the CAIW covers 65.6% of Mongolia’s surface area and receives only 12% of the annual precipitation [19].

2) The AOW has a highly connected network of sub-drainages that exit Mongolia by draining to the north, providing about 50% of the water inflow to Russia’s Lake Baikal (60% of Lake Baikal’s watershed), while the CAIW has 11 disconnected sub-drainages believed to be remnant of Tertiary and Quaternary drainages [20]. Therefore, connectivity as expressed in this study represents the connectivity between sub-drainages within a watershed and via the stream network. This connectivity contrasts one watershed having one major stream network of sub-drainages (i.e., the AOW) and one watershed having 11 disconnected sub-drainages (i.e. the CAIW), 4 of these draining the Lake Uvs basin and the Altai mountain range were sampled for this study (Figure 1A, 1B). These 4 sub-drainages enclose the majority of streams of the CAIW.

3) The AOW is located at the boundary of the Siberian cryosphere, therefore having the highest permafrost extent in Mongolia (Figure 1B), while the permafrost is less expanded in the CAIW and is mainly restricted to high altitudes.

The database for this study was obtained from 114 streams (37 from the AOW and 77 from the CAIW) sampled by the Mongolian Aquatic Insect Survey [http://clade.ansp.org/entomology/mongolia/] during multiple expeditions carried out in Mongolia from 2003 to 2010. Each stream site was sampled once during the 8 years timeframe. Sample sites in the AOW had a median elevation of 1432 m a.s.l. (minimum: 702 m, maximum: 2215 m) and sample sites of the CAIW had a median elevation of 2029 m a.s.l. (minimum: 955 m, maximum: 3087 m). This difference in altitudes of the stream-sites can be considered as an uncontrolled bias as high altitude streams from the AOW were scarce overall in the watershed and the majority were dry during the sampling expeditions. A recent sharp decrease of the glaciers in the Hangai Mountains may have contributed to the lack of summer-flow in the headwater streams of this mountain range (JG personal observation). The frost-free period in Mongolia extends on average from late May to late August; therefore, all the sampling expeditions were carried out in July.

At all sites the invertebrate communities were sampled following a kick sampling protocol using a 500 μm net mesh size and complying with the US EPA Rapid Bioassessment Protocol [21]. Invertebrates were sorted in the lab under dissection microscopes and identified using regional identification keys [22-27].

### Effects induced by climate change

Temperature, precipitation and humidity patterns, snow-cover decrease, glaciers melting and permafrost thaw are considered to be the main abiotic elements strongly affected by climate change in Mongolia. A substantial increase of the mean temperature by 1.6°C was observed over a 60-year period in Mongolia (1940-2001) [28]. In addition to the increase of temperature, patterns of rainfall are considerably affected, causing locally diluvial runoffs events. For example, Li *et al*. [29] found that the relative area affected by severe moisture deficit or excess increased dramatically over Mongolia since 1970, which can be related to the observed events of localized extreme rainfall. This increase of temperature and the change in moisture patterns are affecting the snow-cover depth, and a negative correlation between snow-cover depth and temperature is occurring over wide areas from November to March [30]. In addition to the changes in seasonal climatic elements, climate change is affecting the long-lasting cryologic formations in Mongolia. Shrinkage of Mongolian glaciers has been confirmed by Kadota and Gombo [31] and the fragile state of the Mongolian permafrost [32] suggests major upcoming changes.

These changes will be directly affecting the stream invertebrate communities. For instance, water temperature and thermal regimes regulate invertebrate population growth, population dynamics and overall niche expansion [33]. Increases in the rates of glacial melt and permafrost thaw regulate surface runoffs and water infiltration, consequently affecting stream discharge regime and therefore the structure of invertebrate communities [see, [34,35]. Additionally, permafrost thaw and diluvial runoffs events are expected to increase the input of eroded particles to the stream system, consequently increasing the siltation of the substrate and affecting the stream water biogeochemistry [8] toward a general eutrophication of the stream system.

For these reasons, all biological traits deemed to be sensitive to or to describe affinities to temperature, disturbance, substrate structure, trophic status and eutrophication (Table 1) were used in this paper to describe the stream macroinvertebrate metacommunities of Mongolia.

**Table 1:** Categories of biological traits used to describe macroinvertebrates life cycles and their affinities to temperature, disturbance, substrate structure, trophic status and eutrophication

**Traits**	**Categories**	**Expected Mechanisms**
Maximal size (cm)	0.25-0.5	Disturbance due to frequent discharge fluctuation and siltation will favor small size invertebrates having a higher resilience capacity
	0.5-1	
	1-2	
	2-4	
	4-8	
Dispersal	Aquatic passive	Passive dispersal is expected to be more common when stream physical connectivity is high
	Aquatic active	
	Aerial passive	
	Aerial active	
Lifecycle duration	≤ 1 year	Taxa having a short development cycle have a higher resilience capacity and therefore are more able to maintain populations in frequently disturbed environments
	> 1 year	
Potential number of life cycles per year	< 1	Populations having more than one cycle per year have a higher resilience capacity adapted to frequently disturbed environments
	1	
	> 1	
Current velocity (cm.s^-1^)	Null	An increase in water discharge will favor organisms preferring medium to fast current velocities
	Slow (<25)	
	Medium (25-50)	
	Fast (>50)	
Trophic status	Oligotrophic	An increase of nutrient release by permafrost thaw will favor mesotrophic and eutrophic taxa
	Mesotrophic	
	Eutrophic	
Temperature preferendum	Cold (< 15°C)	Climate change will negatively affect the cold stenothermic taxa
	Warm (> 15°C)	
	Eurythermic	
Saprobity	Xenosaprobic	In a eutrophic ecosystem, meso- and polysaprobic taxa are more likely to occur
	Oligosaprobic	
	α-mesosaprobic	
	β-mesosaprobic	
	Polysaprobic	
Substrate preferendum	Cobble	An increase of suspended particles and substrate siltation will increase the percentage of taxa adapted to fine-grain substrates
	Gravel	
	Sand	
	Silt	
	Macrophytes	
	Microphytes	
	Roots	
	Detritus	
	Mud	
Feeding habits	Deposit-feeder	Eutrophication of the stream system will increase the percentage of deposit-feeder and filter-feeder taxa
	Shredder	
	Scraper	
	Filter-feeder	
	Predator	

The expected mechanisms column indicates how these traits may affect the macroinvertebrate communities under a global warming scenario.

### Data analysis

#### Community structure analysis

The total number of taxa per watershed constitutes gamma diversity. Since the numbers of sampled stream-sites per watershed were not equal, richness accumulation curves [36] were used to estimate total richness and to reduce bias from differences in the sampling effort. Accumulation curves were produced using the model Chao1 [37] available in Estimates 8.2.0 http://purl.oclc.org/estimates.

Beta diversity, between stream diversity, was used to quantify the level of distinctness of the stream invertebrate communities at the watershed scale. Beta diversity values between watersheds were compared using *t*-test, (significance threshold 5%), followed by an analysis of nestedness to assess the impact of rare species on the community structure. Nestedness represents the degree to which small assemblages of taxa are subsets to successively larger assemblages and can be viewed as a region-wide outcome of a taxa pool being filtered by site-specific environmental constraints [12,38]. To quantify the community nestedness across the two watersheds we used a nestedness temperature calculator. Calculations were done on binary-matrices for each watershed (159 taxa x 37 stream-sites and 159 taxa x 77 stream-sites), where a temperature value (*T*) of 0° shows a perfectly nested distribution (rare taxa are in diversity rich sites) and a *T* of 100° shows a random distribution of the taxa. Matrix temperatures were tested for statistical significance using a Monte-Carlo test (comparison to 999 randomly simulated matrices). Nestedness analysis was performed using NESTEDNESS software [39].

We excluded all rare taxa (i.e., taxa that occurred once) and performed a Correspondence Analysis (CA) to describe the structure of the metacommunities. The CA was conducted on a reduced matrix of 100 taxa (59 taxa were removed) and 114 stream-sites. Prior to the CA, data were transformed to logarithmic values (ln(x + 1)) in order to reduce and normalize the variance.

#### Analysis of guild structure

Taxa were assigned to particular biological trait categories (Table 1) according to the database developed by Tachet *et al.*[40]. The database was preferred for two reasons: 1) a higher similarity between European and Central Asian fauna when compared to other available datasets (e.g. North American fauna), and 2) the fuzzy coding used in this database is expected to minimize potential analysis errors due to phenotypic plasticity between Asian and European genotypes. Minor additions to the database were made based on personal communications from taxonomic experts mainly for Plecoptera.

Biological traits were available for 69 of the 100 taxa that occurred more than once in our data set. Therefore, the analysis was computed on a matrix of 69 taxa and 114 stream-sites. Ten biological traits described in 43 categories were included in the analysis (Table 1), and the affinities of taxa for the categories were coded in a fuzzy coding of frequency distribution [15]. Fuzzy coding, as described by Chevenet et al. [41], allows taxa to exhibit categories of each biological trait to different degrees. This takes account of variation in trait expression between life stages and between individuals at each life stage [42], and as we mentioned above, should minimize errors due to phenotypic plasticity. We analyzed the frequencies of the categories by a Fuzzy Correspondence Analysis (FCA) [41]. The categories were weighted by the log-transformed (ln(x + 1)) abundance of each taxon at a site subsequent to a CA (69 taxa and 114 stream-sites). Finally, the stream-sites were clustered per watershed for an easier visualization of the results.

All multivariate analyses (CA and FCA) were computed using the ade4 package in R [43].

## Results

### Community structure

**Figure 2:**
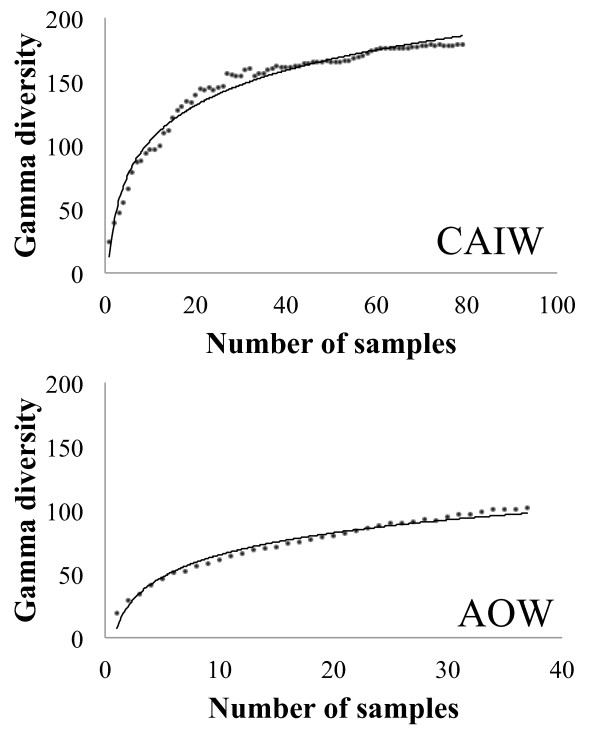
**Gamma diversity accumulation curves showing the saturation curves (bold line) of taxa diversity in both watersheds (CAIW and AOW are the same abbreviations in Figure**1**).**

In total 159 taxa were identified from 114 stream-sites, including 125 different genera (Table 2). Gamma diversities were 80 for the AOW (37 samples) and 136 for the CAIW (77 samples). The richness accumulation curves corroborated these gamma diversity values and showed near-saturation values of 100 and 178 stream samples in the AOW and the CAIW, respectively (Figure 2). Fifty seven taxa were common to both watersheds, while 23 taxa were exclusive to the AOW, constituting 28.7% of the gamma diversity of this watershed; and 79 taxa were exclusive to the CAIW constituting 58% of the gamma diversity of this watershed. The differences in the contribution of these exclusive taxa to the overall richness of the watershed are a major difference between these two watersheds. Beta diversity values were also significantly different (*p* < 0.001) between the two watersheds. Values were higher at the CAIW having a median value of 124, while the median value was 68 in the AOW. The nestedness analysis showed also that communities of the CAIW had a higher nested structure than communities of the AOW (*T* = 15.23 ° and 27.07 °, respectively; *p* <0.001). Therefore, these 3 results show that the CAIW has a higher heterogeneity of its stream communities when compared to the AOW.

**Table 2:** **Taxa list and occurrences in the two watersheds, with *n *indicating the number of stream-sites sampled per watershed**

		**AOW**	**CAIW**
		***n* = 37**	***n* = 77**
**Annelida**			
	Clitellata	-	4
	Oligochaeta	15	29
	Hirudinea	3	3
**Arthropoda**			
**Insects**	**Coleoptera**		
	*Agabus* sp. Leach, 1817	-	5
	*Berosus* sp. Leach, 1817	1	-
	*Bryothinusa* sp. Casey, 1904	-	1
	Dytiscidae	3	3
	*Echinocnemus* sp. Schonherr, 1843	-	1
	*Enochrus* sp. Thomson, 1859	-	1
	Gyrinidae	-	1
	Haliplidae	-	1
	*Helophorus sibiricus* Motschulsky, 1860	-	1
	*Helophorus* sp. Fabricius, 1775	-	2
	*Hygrotus* sp. Stephens, 1829	-	8
	*Laccophilus* sp. Leach, 1815	-	1
	*Oreodytes sanmarkii* Sahlberg, 1826	-	2
	*Oreodytes* sp. Seidlitz, 1887	-	6
	Staphylinidae	-	1
	**Diptera**		
	*Aedes* sp. Meigen, 1818	-	1
	*Agathon* sp. Rodor, 1890	-	9
	*Antocha* sp. Osten Sacken, 1860	-	1
	*Atherix* sp. Meigen, 1803	1	-
	*Bezzia* sp. Kieffer, 1899	-	1
	*Chelifera* sp. Macquart, 1823	-	1
	Chironomidae	35	53
	*Chrysops* sp. Meigen, 1803	-	1
	*Clinocera* sp. Meigen, 1803	-	3
	*Corynoptera* sp. Winnertz, 1867	-	1
	*Deuterophlebia* sp. Edwards, 1922	-	13
	*Dicranota* sp. Zetterstedt, 1838	14	33
	*Dixa* sp. Meigen, 1818	3	-
	*Dixella* sp. Dyar & Shannon, 1924	-	2
	*Ephydra* sp. Fallen, 1810	-	1
	*Eristalis* sp. Latreille, 1804	-	3
	*Gymnopais* sp. Stone, 1949	-	3
	*Helius* sp. Lepeletier & Serville, 1828	1	-
	*Hexatoma* sp. Latreille, 1809	4	2
	*Limnophila* sp. Macquart, 1834	1	2
	*Lispe* sp. Latreille, 1796	1	-
	*Metacnephia* sp. Crosskey, 1969	4	25
	Muscidae	-	4
	*Oreogeton* sp. Schiner, 1860	-	2
	*Pericoma* sp. Walker, 1856	-	2
	*Philorus* sp. Kellogg, 1903	-	1
	*Prosimulium* sp. Roubaud, 1906	3	25
	*Rhabdomastix* sp. Alexander, 1911	-	1
	*Rhaphium* sp. Meigen, 1803	-	3
	Simuliidae	1	-
	*Simulium* sp. Latreille, 1802	9	30
	*Sulcicnephia* sp. Rubtsov, 1971	11	20
	*Tabanus* sp. Linnaeus, 1758	1	2
	*Tipula (Arctotipula)sacra* Alexander, 1946	-	1
	*Tipula (Arctotipula)* sp.	11	23
	*Tipula (Arctotipula)* sp. 1	-	5
	*Tipula (Savtshenkia)* sp.	1	-
	*Tipula (Tipula)* sp.	-	1
	*Tipula* sp. Linnaeus, 1758	-	9
	Tipulidae	-	1
	*Wiedemannia* sp. Zetterstedt, 1838	1	6
	**Ephemeroptera**		
	*Acentrella* sp. Bengtsson, 1912	5	54
	*Ameletus inopinatus* Eaton, 1887	-	3
	*Ameletus* sp. Eaton, 1885	8	26
	*Baetis* sp. Leach, 1815	28	69
	*Baetopus* sp. Keffermuller, 1960	2	12
	*Baetopus trishae* Waltz, 2002	-	3
	*Caenis* sp. Stephens, 1835	2	3
	*Cinygmula* sp. McDunnough, 1933	8	32
	*Drunella* sp. Needham, 1905	14	6
	*Ecdyonurus* sp. Eaton, 1868	12	25
	*Epeorus pellucidus* Kluge, 1989	-	3
	*Epeorus* sp. Eaton, 1881	6	11
	*Ephemera* sp. Linnaeus, 1758	1	-
	*Ephemerella lenoki* Tshemova, 1952	-	1
	*Ephemerella* sp. Walsh, 1862	18	30
	*Ephoron nigridorsum* Tshernova, 1934	-	2
	*Ephoron* sp. Williamson, 1802	3	5
	*Heptagenia* sp. Walsh, 1863	-	5
	Heptageniidae	-	2
	*Isonychia* sp. Eaton, 1871	1	-
	*Leptophlebia* sp. Westwood, 1840	4	-
	*Metretopus* sp. Eaton, 1901	10	-
	*Parameletus* sp. Bengtsson, 1908	-	1
	*Procloeon* sp. Bengtsson, 1915	4	4
	*Rhithrogena* sp. Eaton, 1881	12	25
	*Serratella* sp. Edmunds, 1959	20	59
	*Siphlonurus* sp. Eaton, 1868	10	34
	**Hemiptera**		
	*Arctocorisa* sp. Wallengren, 1894	-	2
	*Callicorixa* sp. White, 1873	-	1
	*Cenocorixa* sp. Hungerford, 1948	-	1
	Corixidae	2	4
	Gerridae	1	1
	*Gerris* sp. Fabricius, 1794	-	1
	*Glaenocorisa* sp. Thomson, 1869	-	1
	*Micronecta* sp. Kirkaldy, 1897	-	1
	*Saldula* sp. Van Duzee, 1914	-	1
	*Teloleuca* sp. Reuter, 1912	-	1
	**Megaloptera**		
	*Sialis* sp. Latreille, 1802	10	-
	**Odonata**		
	*Lestes* sp. Fabricius, 1798	-	2
	*Leucorrhinia* sp. Burmeister, 1839	-	1
	*Ophiogomphus* sp. Fourcroy, 1785	1	-
	**Plecoptera**		
	*Agnetina* sp. Klapálek, 1907	6	4
	*Alaskaperla* sp. Stewart & DeWalt, 1991	1	-
	*Allocapnia* sp. Claassen, 1928	-	1
	*Amphinemura* sp. Ris, 1902	1	1
	*Arcynopteryx compacta* McLachlan, 1872	-	2
	*Arcynopteryx* sp. Klapálek, 1904	-	22
	Chloroperlidae	1	-
	*Diura* sp. Billberg, 1820	-	8
	*Isoperla* sp. Banks, 1906	-	29
	*Leuctra* sp. Stephens, 1836	1	-
	*Megarcys* sp. Klapálek, 1912	-	1
	*Mesocapnia* sp. Rauser, 1969	-	5
	*Nemoura* sp. Latreille, 1796	-	10
	*Pictetiella asiatica* Zwick & Levanidova, 1971	-	1
	*Skwala* sp. Ricker, 1943	-	32
	*Suwallia* sp. Ricker, 1943	8	27
	*Suwallia teleckojensis* Samal, 1939	-	2
	*Triznaka* sp. Ricker, 1952	-	1
	**Trichoptera**		
	*Agapetus* sp. Curtis, 1834	1	-
	*Anabolia* sp. Stephens, 1837	1	1
	*Apatania* sp. Kolenati 1848	7	13
	*Arctopsyche* sp. McLachlan, 1868	4	2
	*Asynarchus* sp. McLachlan, 1880	12	19
	*Brachycentrus* sp. Curtis, 1834	17	60
	*Brachycercus* sp. Curtis, 1834	7	3
	*Ceraclea* sp. Stephens, 1829	4	1
	*Cheumatopsyche* sp. Wallengren, 1891	1	-
	*Clostoeca* sp. Banks, 1943	5	10
	*Dicosmoecus* sp. McLachlan, 1875	4	20
	*Ecclisomyia* sp. Banks, 1907	7	12
	*Glossosoma* sp. Curtis, 1834	7	4
	*Goera* sp. Stephens, 1829	5	9
	*Homophylax* sp. Banks, 1900	-	1
	*Hydropsyche* sp. Pictet, 1834	2	3
	*Lepidostoma* sp. Rambur, 1842	-	4
	*Limnephilus* sp. Leach, 1815	-	3
	*Micrasema* sp. McLachlan, 1876	2	-
	*Molannodes* sp. McLachlan, 1866	-	1
	*Nemotaulius* sp. Banks, 1906	2	2
	*Oligostomis* sp. Kolentai, 1848	1	-
	*Philarctus* sp. McLachlan, 1880	-	1
	*Potamyia* sp. Robineau & Desvoidy, 1830	1	-
	Psychomyiidae	1	-
	*Rhyacophila* sp. Pictet, 1834	14	19
	*Triaenodes* sp. McLachlan, 1865	-	1
**Other Arthropoda**			
	Cladocera	-	4
	*Gammarus* sp. J. C. Fabricius, 1775	1	7
	Hydracarina	9	23
**Mollusca**			
	Bivalvia	1	-
	*Coretus* sp. Adanson, 1757	1	4
	*Gyraulus* sp. Charpentier, 1837	2	10
	*Lymnaea* sp. Lamarck, 1799	-	1
	Planorbidae	2	3
	*Radix* sp. Montfort, 1810	2	7
	Sphaeriidae	5	1
**Nematomorpha**			
	Gordiidae	-	1
	*Gordius* sp. Linnaeus, 1758	-	3
**Tricladida**			
	*Euplanaria* sp. Hesse 1897	-	2
**Platyhelminthes**			
	Turbellaria	4	7

**Figure 3:**
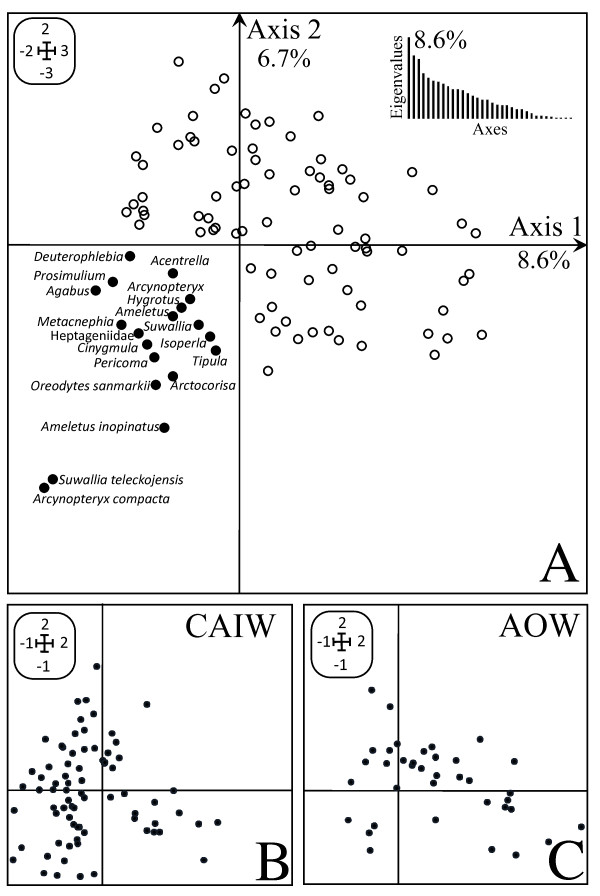
**Plots of the Correspondence Analysis showing the taxa distribution (Figure 3A) and the distribution of samples in the CAIW (Figure 3B) and the AOW (Figure 3C).** Taxa discussed in the manuscript are represented in bold circles in Figure 3A associated to the taxa names.

The first two axes of the CA expressed 15.3% of the variance (Figure 3) with a total inertia of 3.69. The variability explained by the CA is significant considering the high number of taxa included in the computation. The overall distribution of taxa scores showed similar patterns for the communities of the two watersheds. However one assemblage was more strongly associated with the CAIW. This assemblage of macroinvertebrates was composed mainly of cold headwater taxa (*Ameletus inopinatus*, *Ameletus* sp., *Cinygmula* sp., *Deuterophlebia* sp.) and taxa adapted to intermittent stream flow (*Agabus* sp., *Arctocorisa* sp., *Hygrotus* sp, *Oreodytes sanmarkii*). The other taxa of this assemblage were mainly trout-stream type taxa, like *Suwallia* sp., *Suwallia teleckojensis*, *Isoperla* sp., and *Acentralla* sp. While 28 stream-sites out of 77 in the CAIW (≈ 36%) were correlated to this assemblage (Figure 3B), only four stream-sites out of 37 in the AOW (≈ 10 %) were correlated to this assemblage (Figure 3C).

### Analysis of ecological guilds’ structure

**Figure 4:**
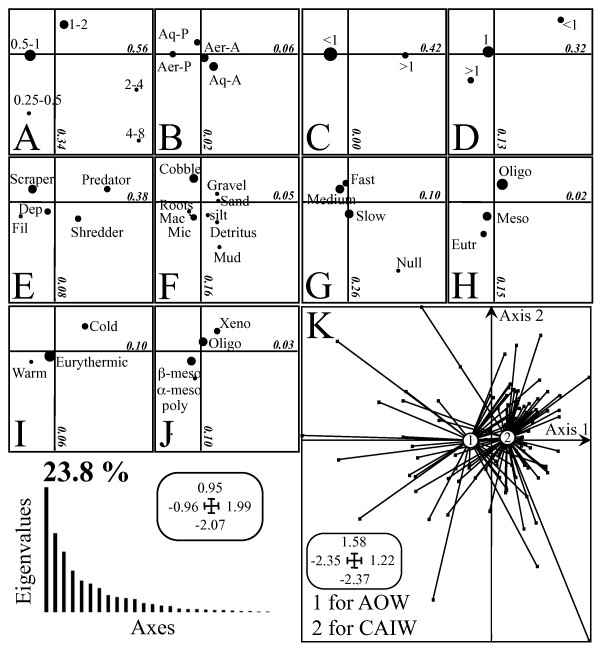
**Plots of the Fuzzy Correspondence Analysis showing the distribution of the different categories of each biological trait (Figure 4A to J), these same categories are listed in Table**1**.** Figure 4**K** shows the distribution of the stream-sites clustered per watershed (1 for AOW and 2 for CAIW). In Figures 4A to J, the size of the black bold circles representing the traits’ categories indicates the weight of these categories in the analysis. Plot **A** is for maximal size, plot **B** for dispersal (Aq-P for aquatic passive dispersal, Aq-A for aquatic active dispersal, Aer-P for aerial passive dispersal, Aer-A for aerial active dispersal), plot **C** for life cycle duration, plot **D** for potential number of life cycles per year, plot **E** for feeding habits (Dep and Fil are for Deposit- and Filter-feeder, respectively), plot **F** for substrate preferendum (Mac and Mic are for Macrophyte and Microphyte, respectively), plot **G** for current velocity, plot **H** for trophic status (Oligo, Meso and Eutr are for Oligotrophic, Mesotrophic and Eutrophic, respectively), plot **I** for temperature preferendum, and plot **J** for saprobity (Xeno, Oligo, α-meso, β-meso, Poly are for Xenosaprobic, Oligosaprobic, α- mesosaprobic, β-mesosaprobic and Ploysaprobic, respectively).

The FCA ordination of guild structure summarizes 38.8% of the overall variability with a total inertia of 0.88 (Figure 4). The variables having the highest correlations of variables to axis 1 were maximal size (Figure 4A), life cycle duration (Figure 4C), potential number of life cycles per year (Figure 4D), feeding habits (Figure 4E), current velocity (Figure 4G) and temperature preferendum (Figure 4I). Those having the highest correlations of variables to axis 2 were maximal size (Figure 4A), current velocity (Figure 4G), substrate preferendum (Figure 4F) and trophic status (Figure 4H). Thus, axis 1 contrasts small body size (see Figure 4A, < 1cm), multivoltine scrapers and deposit feeders (Figure 4C, 4E), giving lower site scores, with large bodied uni- or semivoltine predators and shredders. Axis 2 represents a gradient in tolerance of taxa to nutrient enrichment (Figure 4H) and saprobicity (Figure 4J): taxa tolerant of eutrophication conditions contribute to low scores on axis 2 whereas oligotrophic taxa are associated with high scores. In addition, axis 2 contrasts the substrate preferences of taxa (Figure 4F). Dispersal, which plays a key role in modeling responses to climate change, is also associated with axis 1 (Figure 4B): taxa with active aquatic and aerial dispersal behavior were associated with high scores on axis 1. The temperature preferendum categories plot (Figure 4I) shows that the majority of the taxa were eurythermic, and to a less extent taxa preferring warm temperature (negative side of axis 1) and taxa preferring cold temperature (positive side of axis 1).

The majority of the categories were distributed along axis 1 and the stream-sites from the two watersheds were distinctly clustered along the same axis. Stream-sites from the AOW were clustered on the negative side of axis 1 and therefore have higher correlations with the trait categories on this side of axis 1. The stream-sites from the CAIW had higher correlations to the trait categories located on the positive side of axis 1and were opposed to those from the AOW.

## Discussion

### Communities and ecological differences between the two watersheds

The differences in the structure of communities between the two watersheds are the result of geological history and natural variation in the riverine landscape. No dams or channelization has occurred in the Mongolian rivers, so the lower biodiversities (both gamma and beta diversities) of the AOW cannot be attributed to anthropogenic homogenization across drainages [sensu, [44]. A prediction, complying with the niche theory [45], states that higher biodiversity in the CAIW could result from higher diversity of habitats in the watershed. The CA outcome shows that the community of the CAIW is distinguished by having taxa assemblages of intermittent streams and cold headwater streams. These assemblages included some remarkable taxa found only in high altitude glacial-fed streams, like the mountain-midge (*Deuterophlebia* sp.) reported previously in glacier streams of the Himalaya and Tian-Shan Mountain ranges [46]. The assemblages of cold headwater streams were found in low-order streams draining the Altai Mountain range that stretches from northwestern Mongolia (see Figure 1) into Kazakhstan and Russia. Similar cold-water streams were not encountered in the high altitudes of the Hangai Mountains of the AOW, where glaciers were sparse and many high-altitude streams were dry during the summer sampling expeditions.

Higher beta diversity values and lower nestedness temperature in the CAIW confirm our initial hypothesis that lower sub-drainages connectivity would support higher distinctiveness of communities. The importance of dispersal through the riverine network was also indicated by guild structure analysis. The disconnected sub-drainages of CAIW prevent passive dispersal via the riverine network (e.g. downstream drift dispersal); consequently both aquatic and aerial passive dispersals were less relevant to the structure of the communities in this watershed (Figure 4B). In contrast, passive dispersals via aquatic and aerial means were more relevant to the community structure of the AOW due to a higher physical linkage between the streams. The dendritic connections of sub-watersheds are exploited by actively dispersing fauna such as fishes, as has been observed at large scale in the Mississippi-Missouri drainage [see, [47]. While dispersal in stream-insect communities was always considered to be primarily influenced by local environmental factors [48], and to a lesser extent by a combination of local and regional factors [49]. Our results highlight the importance of stream network connectivity in constraining dispersal of aquatic insects through the larger watershed.

A higher connectivity of sub-drainages also means a higher connectivity of vegetated riparian buffers that can provide sheltered habitat corridors for the dispersal of aquatic imagos. In Mongolia, such dispersal routes (i.e. following the network of riparian vegetation) can play an important role due to the structure of the Mongolian landscape. The lack of dense vegetation on the xeric Mongolian short grass steppe and a windy environment may restrict swarming activities of aquatic imagos to the riparian vegetation. This hypothesis corroborates the outcome of Petersen *et al.*[50] finding that the stream corridor, including the riparian vegetation, is the main habitat for active aquatic imagos. Consequently a higher connectivity in the riparian vegetation between sub-drainages contributes also to increasing the aerial dispersal of aquatic insects via the stream corridor routes. This interpretation is supported by the guild structure analysis, where aerial active dispersal was correlated to both aquatic passive and active dispersals, since both aquatic dispersals are dependent of aquatic corridors linkage between the sub-drainages.

### Expected changes due to global warming

The consequences of global warming on the stream communities in Mongolia will be dependent on 1) their current composition, and 2) the magnitude of the thermal and hydrological changes. Macroinvertebrates in streams of the AOW have more traits providing better resistance and resilience to disturbance, and many taxa there are typified by r-selected traits. Correspondingly, the AOW community has more taxa with r strategy, having a higher occurrence of r-selection traits [51]. Small body size, short life cycle (i.e. < one year duration) and multiple generations per year are the main traits that characterize community of the AOW. The community of the CAIW has more taxa with the K strategy, lifecycle with traits reflecting high biotope stability. In addition, the trait analysis differentiated the communities of these two watersheds based on their trophic status. The community of the AOW shows a higher tolerance to eutrophication with more scrapers and filter-feeders (Figure 4E), and meso- and polysaprobic taxa (Figure 4J), found in meso- and eutrophic conditions (Figure 4H). Eurythermy describes the majority of the taxa in both watersheds, whereas cold-water taxa occurred mainly in the CAIW.

Beyond the functional differences between communities of the watersheds, remarkable differences in the extent of permafrost (see Figure 1) and the amount of rainfall per surface-area unit received by each watershed suggest different magnitudes for the impacts of climate change per watershed. More permafrost thaw in the AOW coupled to localized intense rainfall will be translated into higher runoff and an increasing input of soil particles into the drainages. Therefore, even though the assemblage of this watershed seems to be resilient to disturbances and slight eutrophication, selection for taxa having greater adaptation to eutrophication is likely to occur in the future. Taxa intolerant to higher temperatures and siltation in the AOW are likely to decline although the higher opportunity for dispersal may moderate the probability of population loss at the watershed scale.

A different response to climate change is expected in the CAIW. The isolated structure of sub-watersheds and the K-selected traits would suggest higher vulnerability to major climatic changes. Following an initial increase in the summer discharge affected by glacial meltwaters, the longer-term effect on stream will be an increase of intermittent-flow conditions favoring r-selected traits and therefore favoring a community adapted to intermittency. Eutrophication associated with permafrost thaw is expected to have limited impact compared to increased flow and higher water temperatures which will have greater impact on the cold stenothermal taxa occurring in the headwaters of the Altai Mountain range. The loss of these taxa would result in a major reorganization of the community composition in mountain streams of the CAIW.

As climate change affects these watersheds differently, we would expect species replacement and a shift in the community’s structures to lead to a greater divergence between the metacommunities of the CAIW and the AOW. The AOW community is expected to exhibit increased tolerance to eutrophication, while the CAIW community is expected to shift toward lower diversity and greater distinctness of local assemblages (sensu, sub-drainages’ assemblages) due to local extinction and reduced colonization by better adapted taxa. Conservation planning must incorporate the connected nature of drainages, a key factor for the conservation of freshwater biodiversity [52] when understanding how climate change will affect the freshwater biodiversity in Mongolia. In addition, Mongolia is currently witnessing increases in mining and livestock production [see, [53] which also threaten the integrity of its river ecosystems. We recommend that conservation plans should account for major differences in the beta diversity, dispersal and resilience capacity of stream biota in these two major watersheds.

## **Competing interests**

The authors declare that they have no competing interests.

## **Author’s contributions**

AM participated to the field expedition in 2010, compiled data, ran the analysis and wrote the paper. JG served as PI on the two grants supporting this work and led the expeditions of the Mongolian Aquatic Insect Survey. All authors read and approved the final manuscript.
